# Comparative Chemical Analysis and Bioactive Properties of Aqueous and Glucan-Rich Extracts of Three Widely Appreciated Mushrooms: *Agaricus bisporus* (J.E.Lange) Imbach, *Laetiporus sulphureus* (Bull.) Murill and *Agrocybe aegerita* (V. Brig.) Vizzini

**DOI:** 10.3390/ph17091153

**Published:** 2024-08-31

**Authors:** Jovana Petrović, Jasmina Glamočlija, Danijel D. Milinčić, Ana Doroški, Steva Lević, Slađana P. Stanojević, Aleksandar Ž. Kostić, Dušanka A. Popović Minić, Bojana B. Vidović, Ana Plećić, Viktor A. Nedović, Mirjana B. Pešić, Dejan Stojković

**Affiliations:** 1 Institute for Biological Research, Siniša Stanković“—National Institute of the Republic of Serbia, University of Belgrade, Bulevar Despota Stefana 142, 11108 Belgrade, Serbia; jovana0303@ibiss.bg.ac.rs (J.P.); jasna@ibiss.bg.ac.rs (J.G.); 2 Institute of Food Technology and Biochemistry, Faculty of Agriculture, University of Belgrade, Nemanjina 6, 11080 Belgrade, Serbia; danijel.milincic@agrif.bg.ac.rs (D.D.M.); ana.doroski@agrif.bg.ac.rs (A.D.); sladjas@agrif.bg.ac.rs (S.P.S.); akostic@agrif.bg.ac.rs (A.Ž.K.); dusanka.popovic@agrif.bg.ac.rs (D.A.P.M.); ana.bjekovic96@gmail.com (A.P.); vnedovic@agrif.bg.ac.rs (V.A.N.); 3 Faculty of Pharmacy, University of Belgrade, Vojvode Stepe 450, 11221 Belgrade, Serbia; bojana@pharmacy.bg.ac.rs

**Keywords:** mushroom extracts, chemical characterization, antioxidant agents, ATR-FTIR, antimicrobial agents, anti-inflammatory agents, wound-healing properties

## Abstract

Herein we describe the antioxidant, antimicrobial, antibiofilm, anti-inflammatory and wound-healing potential of aqueous and polysaccharide extracts from three widely appreciated mushrooms: *Agrocybe aegerita*, *Laetiporus sulphureus* and *Agaricus bisporus*. Moreover, we present their detailed phenolic, polysaccharide and protein profiles and ATR-FTIR spectra. The study found that polysaccharide extracts (PEs) from mushrooms had higher total and *β*-glucan levels than aqueous extracts (AEs), with *A. aegerita* showing the highest content. *L. sulphureus* had a higher total protein content, and *A. aegerita* AE had the highest phenolic content. Our results indicate that all the tested extracts have high potential regarding their bioactive properties, with *A. aegerita* being the most promising one. Namely, the antibacterial activity assay showed that the development of the skin-infection-causing agent, *Staphylococcus aureus*, was inhibited with a minimal inhibitory concentration of 4.00 mg/mL and minimal bactericidal concentration of 8.00 mg/mL, while the results regarding wound healing showed that, over the course of 24 h, the *A. aegerita* extract actively promoted wound closure in the HaCaT keratinocyte cell line model. The anti-inflammatory activity results clearly showed that when we used *S. aureus* as an inflammation-inducing agent and the *A. aegerita* aqueous extract in treatment, IL-6 levels reduced to the level of 4.56 pg/mL. The obtained data suggest that the tested mushroom extracts may serve as a source of bioactive compounds, with potential applications in the cosmeceutical, pharmaceutical and food industries. Furthermore, potential skin preparations carefully crafted with mushroom extract may help restore the skin’s barrier function, decrease the probability of staph infections and minimize skin irritation.

## 1. Introduction

Thanks to the globalization process, the world is literally on our plate. What is available in our cuisine has changed dramatically over time, and today we have high-quality food ingredients available all year round. Yet, somehow as human species, we have more disease incidence than ever. People do tend to wish for better dietary habits, but due to a restless lifestyle, most of them just do not have the time to address it. With respect to this, some of the forgotten ideas regarding the use of a balanced and healthy diet in order to achieve optimal health have been re-evaluated. In line with this, so as to achieve optimal health via balanced nutrition, one should not deprive oneself of their favorite food; moreover, eating healthy should be easy and affordable. Herein is where the market for the development of food products fortified with extra nutrients, vitamins, minerals, fiber and protein appears. Wild growing and cultivated mushrooms have emerged as versatile and powerful ingredients to be incorporated into various food products due to their favorable nutritional profile and the presence of multiple bioactive compounds that show antimicrobial, antioxidant, antitumor and immunomodulatory properties [1,2,3,4]. Their macronutrient profile consists mainly of carbohydrates, proteins and a low amount of total lipids, among which polyunsaturated fatty acids are highly abundant. In addition to nutrients, fruiting bodies of mushrooms are also a significant source of compounds such as lectins, terpenoids and phenolics, which do not contribute to dietary requirements but add to the beneficial health effects [5]. This type of dietary outline suggests the consumption of mushrooms on a daily basis, but due to their limited seasonal and geographical availability, they should be incorporated into products that we can consume continuously throughout the year.

Herein, we conducted comprehensive chemical analyses and evaluated various aspects of bioactive properties using three widely consumed mushrooms: *A. aegerita*, *L. sulphureus* and *A. bisporus*. *A. aegerita*, aka chestnut mushroom, is highly valued among consumers, especially in Italy where it is also commercially cultivated. Along with its wide culinary use, it has high nutritional value and has been utilized as a potent antioxidant agent and for its anti-inflammatory properties [6]. Given that a high protein content has been identified in its fruiting bodies (25–30%), *A. aegerita* has been recommended in dietary regimens where significant protein intake is necessary. Certain bioactive properties have been demonstrated for the *A. aegerita* methanolic extract as well, including antioxidant and antimicrobial. Due to its high nutritional value, this mushroom is a highly valuable ingredient in the food industry, particularly to people seeking to consume nutrient-rich food [7,8]. As for *L. sulphureus*, it has been extensively explored in recent years due to its ability to produce various bioactive metabolites with potent biological properties including minerals, polyunsaturated fatty acids, lectins, phenolics, terpenes and many others. In folk medicine, it has been known to alleviate symptoms such as cough, rheumatism, gastric cancer, etc., whereas its use in conventional therapy must be further explored [9]. Cultivated *A. bisporus* has been extensively explored for its chemical constituents and bioactive properties. The obtained results suggest it is a rich source of glucans, sterols and vitamin D, which act as immune enhancers, but they are also highly appreciated for their gastroprotective features. However, further research is needed in order to use it in clinical practice [10]. 

Even though many bioactive properties of *A. aegerita*, *L. sulphureus* and *A. bisporus* have been demonstrated so far, this study aims to characterize their aqueous and polysaccharide extracts in terms of their phenolic, protein and glucan content as well as ATR-FTIR spectra, with a more in-depth polypeptide composition and phenolic profile analysis of mushroom extracts using UHPLC–QToF-MS. Moreover, the comparative analysis of these extracts with respect to their bioactive properties will be evaluated as well, with special emphasis on their antioxidant, cytotoxic, wound-healing, anti-inflammatory, antimicrobial and antibiofilm activities. These will have immense importance in drug development, since they address several health issues highly abundant in the population on a global scale. Given that these are edible mushrooms, their versatile application as a prophylactic agent and therapeutic agent may be considered safe. Furthermore, their clear benefit is that they can be used as functional ingredients to enhance and fortify already available food products, so as to increase their nutritional value or impart certain health benefits. This strategy would allow for the application of high-quality food in cases of an insufficient, excessive or simply imbalanced diet. 

## 2. Results and Discussion

### 2.1. Total α-Glucan and β-Glucan Content in AE and PE

Polysaccharides from fungi have been used extensively in several therapeutic aspects, but their main application is as immunomodulatory and anti-inflammatory agents in diseases such as recurrent infections, or in other cases when an imbalance of the immune system occurs, such as after chemotherapy or after surgery. Glucans, especially the β-type, has also shown protective effects in cardiovascular disease, as well as in diabetes or in maintaining lipid homeostasis [10]. As expected, all PEs showed higher percentages of total and β-glucans in comparison with the AEs, while the highest total and β-glucan content was determined in *A. aegerita* (Table 1). Our samples, especially *A. aegerita*, showed a rather high share of polysaccharides in both the AE and PE (44.28% and 25.47%, respectively), followed by *A. bisporus* (33.37% and 7.32% for the AE and PE, respectively) and *L. sulphureus* (11.47% and 6.92%, for the AE and PE, respectively). Among our samples, the highest content of glucans was identified in the *A. aegerita* PE (34.95%), while *L. sulphureus* had the lowest amount of this group of compounds. Mushroom polysaccharides, especially β-glucans, have been extensively researched in *Agaricus bisporus* and other commercial mushroom species. In these species, β-glucans are found in concentrations ranging from 9 to 33% (dry weight) [11,12], which is in accordance with our current findings. Our results of the comparative investigation indicated that the aqueous extracts of mushroom species were more abundant in terms of β-glucan content when compared to the polysaccharidic extracts.

### 2.2. Total Protein Content of AE and PE

The total protein content of *L. sulphureus* presented in Table 2 showed a significantly higher total protein content in comparison with the other two mushrooms. Proteins were not registered in the polysaccharide extracts. Contrary to our results regarding the total glucan content in the extracts, *L. sulphureus* turned out to be an excellent source of proteins, with 5.62 g BSA/100 g, whereas *A. aegerita* showed the lowest protein content. In general, mushrooms are considered a great source of proteins, with an entire amino acid profile present in their fruiting bodies. The obtained results are in accordance with our previously published results regarding *L. sulphureus* content, though a different method was used. As suggested by Petrović et al. [7], 15.97 g per 100 g of proteins were detected in chicken of the woods via the Macro-Kjeldahl method. As for the protein content in *A. aegerita*, Petrovic et al. [8] determined 6.68 g per 100 g, while Glamočlija et al. [13] showed that the protein content in *A. bisporus* was 10.00 g per 100 g. The lower total protein content of mushroom AEs observed in this study and in previous studies could be attributed to the interference of polysaccharides present in the samples. It has been demonstrated that polysaccharides bind to the Coomassie blue G dye used in the Bradford assay without contributing to the absorbance, leading to an underestimation of the total protein content [14]. Furthermore, phenolic acids (the main phenolic compounds in AEs; see Table 4) interact with proteins, altering their structure and interfering with their properties [15]. This is of the utmost importance since meat, as the main source of protein, is not readily available to the worldwide population. Thus, mushrooms are often regarded as the meat of poverty, since along with their excellent protein content and amino acid profile, some of them can be cultivated using different types of waste, which allows for a circular economy.

### 2.3. Total Phenolic Content (TPC) of AE and PE

The total phenolic content of mushrooms extracts is presented in Table 3. The highest TPC was determined in the AE of *A. aegerita* (23.72 g GAE/100 g), whereas the lowest was recorded in the AE of *L. sulphureus* (0.93 g GAE/100 g). In the PEs of *A. aegerita* and *A. bisporus*, the presence of phenolic compounds was not detected by the applied methodology, as was expected. The presence of phenolic compounds is quite important since this versatile group of compounds has been associated with several bioactive properties, including antioxidant, cholinesterase inhibitory, antimicrobial etc. [16,17].

### 2.4. Protein Profile of Mushroom AE

**Figure 1:**
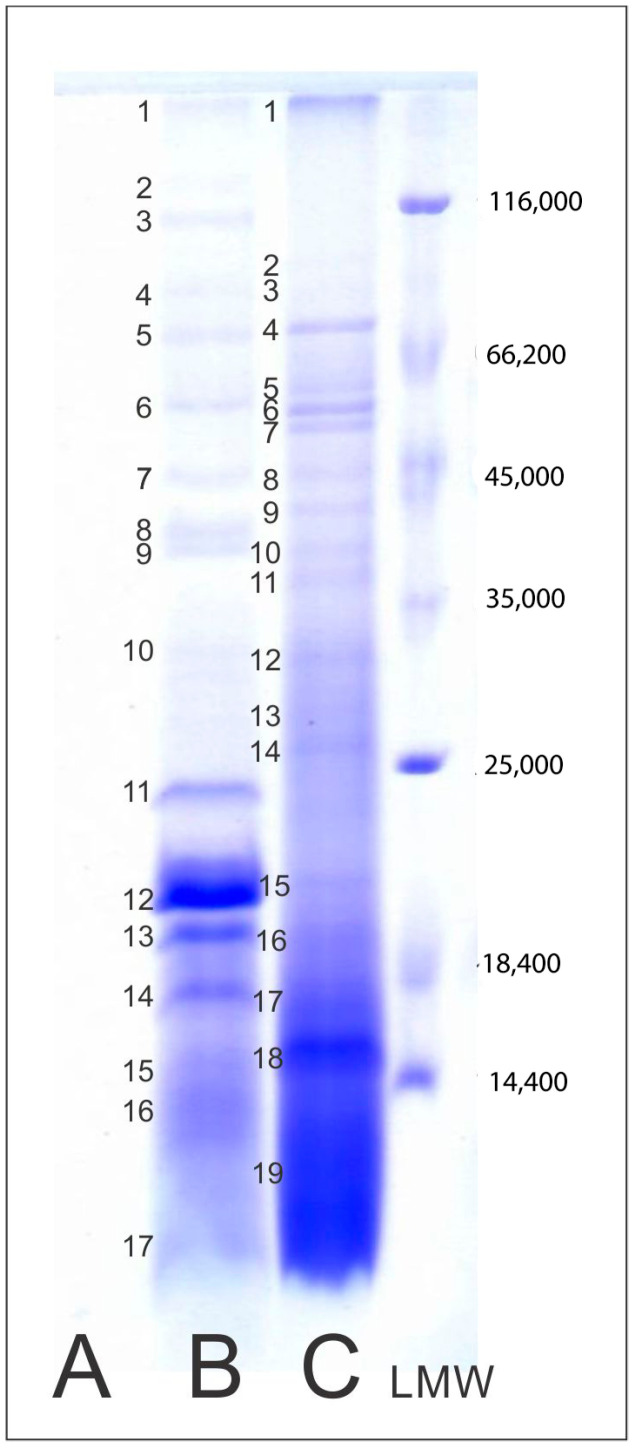
SDS-PAGE electrophoretic patterns of mushroom aqueous extracts of *A. bisporus*, *A. aegerita* and *L. sulphureus*. Abbreviations: (A) AE of *L. sulphureus*; (B) AE of *A. aegerita*; (C) AE of *A. bisporus*; LMW—molecular weight standard. Small numbers on electrophoretic patterns denote bands of specific polypeptides detected in analyzed samples. LMW and the polypeptide composition of detected bands were determined using GelAnalyser 19.1 and are shown in Table S1.

So as to gain greater insight into the polypeptide composition of mushroom aqueous extracts, SDS-R-PAGE analysis in reducing conditions was carried out. As can be seen in the SDS-PAGE patterns (Figure 1B,C), the analyzed *A. aegerita* and *A. bisporus* AEs had completely different polypeptide profiles, with specific bands as unique markers for their authentication, and bioactivities. A great heterogeneity of mushroom protein extracts was also observed by Bauer Petrovska [18] analyzing seven species of Macedonian edible cultured or wild mushrooms. Interestingly, in the SDS-PAGE pattern of the *L. sulphureus* aqueous extract, no bands were visible (Figure 1A). The absence of proteins could be due to protein hydrolysis during the extraction process, which results in shorter polypeptides that cannot be detected on the SDS-PAGE gels used [19]. Refs. [16,17] were also unable to detect protein bands in the SDS-PAGE pattern of *Pleurotus ostreatus* extracts due to the high degree of hydrolysis and high fiber content. The lower degree of hydrolysis in *A. bisporus* strains was also observed by these authors. An excessive amount of loaded sample as well as protease activity during sample preparation may be the cause of poorly defined and smeared bands, as well as the low molecular weight of the *A. bisporus* extract [20].

In the SDS-PAGE patterns of *A. bisporus* and *A aegerita*, in total, 19 and 17 polypeptide bands were detected, respectively (see left-hand side margin of Figure 1 for positions of these polypeptides). These bands were further examined by densitometric analysis (Table S1). The majority of bands could be clearly observed, with a few diffused bands below 25 kDa for both extracts. Due to the easier interpretation of the obtained results, the relative composition of the detected polypeptides was expressed through four polypeptide ranges: <66.2 kDa, 66.2–35 kDa, 35–14.4 kDa and <14.4 kDa, and presented in Table 1. The major polypeptide fractions were confirmed in the MW range of 35–14.4 kDa and <14 kDa, making up > 80% of the soluble polypeptides (Table S1) for both extracts. Finally, the *A. aegerita* extract contained five major bands with MWs of 19.7, 18.9, 17.9, 16.9 and ~14.4 kDa, while the major bands of *A. bisporus* were below 14.4 kDa. Similar results for the *Agaricaceae* family were observed by Bauer Petrovska [18], who registered 13–16 protein bands in SDS-PAGE patterns, dominated by bands of 30 kDa and less.

The presence of proteins in mushroom extracts may be quite important with respect to their ability to contribute to bioactive properties, as was demonstrated by Tehrani et al. [21]. Namely, *A. bisporus* extract showed to have potential to inhibit the growth of pathogenic bacteria, especially MRSA strains. 

### 2.5. UHPLC–QToF-MS Profiling of Mushroom Extracts

Bioactive compounds identified in aqueous and polysaccharide mushroom extracts are presented in Table 4. For these characterizations, untargeted UHPLC–QToF-MS was performed, and biomolecules were identified based on exact mass (*m*/*z*), typical MS fragment differences in retention time (for isomers), available standards and literature data. All identified compounds can be classified into three distinct groups: (i) organic acids and derivatives (12 compounds); (ii) phenolic acids and derivatives (11 compounds); (iii) triterpenoid compounds. Considering the results of the characterization, the differences between the tested samples can be clearly observed (Table 4; malic, citric, pinellic and azelaic acids were detect in all analyzed extracts). In addition, fumaric, succinic and sebacic acids were confirmed in all extracts, except in the polysaccharide extracts of *A. aegerita*, *L. sulphureus* and *A. bisporus*, respectively. The organic acids mentioned have already been detected by other authors in various wild and cultivated mushrooms [22,23,24,25]. The compound recognized as 13-hydroxy-9,11-octadecadienoic acid was also found in all the analyzed extracts. This compound was previously identified and reported by Ye et al. [26] in *Auricularia cornea* mushroom. Other detected derivatives of organic acids identified as pimelic acid, 3-hydroxy sebacic acid, diethyl-3-hydroxyglutarate and 9-hydroxy-13-oxo-10-octadecenoic acid) were confirmed in both *L. sulphureus* extracts. These derivatives were also found selectively in the *A. aegerita* and *A. bisporus* aqueous extracts. Among the phenolic compounds, only phenolic acids and their derivatives were selectively detected in the analyzed extracts. Phenolic compounds were not found in the polysaccharide extracts of *A. bisporus* or *A. aegerita*. On the other hand, the polysaccharide extract of *L. sulphureus* contained several phenolics, including gallic acid and ethyl-homovanillate, which were only detected in this sample. These and other phenolic acids were probably bound to *L. sulphureus* polysaccharides and released during their extraction and precipitation. All aqueous extracts contained phenolic acids, but their presence in the extracts was selective. Most phenolic compounds were identified in the aqueous extracts of *A. aegerita* and *L. sulphureus*, mainly hydroxybenzoic acid, dihydroxybenzoic acid and coumaric acid, and their derivatives. Our results are in agreement with the results of other studies, which showed the most frequent occurrence of these phenolic acid derivatives in various mushroom samples [27,28,29,30,31]. Triterpenoid compound (like maslinic acid) was only confirmed in both *L. sulphureus* extracts. Various triterpenoid compounds were previously reported by Koutrotsios et al. [32] in oyster (*Pleurotus*) mushrooms. The recorded profiles of the present compounds can contribute to the further understanding of the antioxidant and biological properties of the analyzed mushroom extracts, since phenolic acid and its derivatives, as well as organic acids, may contribute to the overall biological activity of the tested mushrooms. As was reported by Alves et al. [16], presence of specific groups is responsible for the antibacterial activity, namely carboxylic acid (COOH), two hydroxyl (OH) groups in the para and ortho positions, as well as a methoxyl (OCH_3_) group in the meta position. The obtained results regarding the presence of phenolic acids is important due to the fact that all three tested mushrooms are edible, and studies conducted so far have shown that the consumption of food rich in phenolic acids can contribute to overall health and well-being. They can especially decrease the incidence of certain diseases due to their multiple demonstrated high activities.

### 2.6. ATR-FTIR Spectroscopy of Mushroom AE and PE

**Figure 2:**
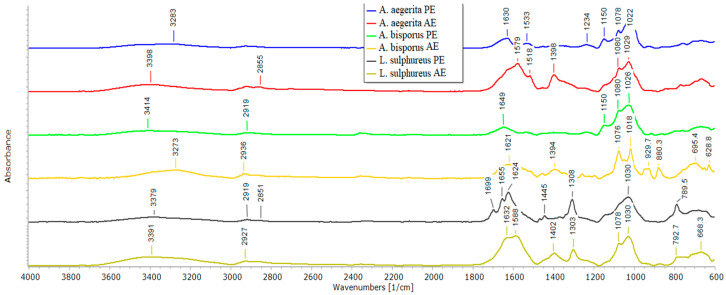
ATR-FTIR spectra of mushroom AE and PE.

The results of the ATR-FTIR analysis are shown in Figure 2. All analyzed spectra show the broad bands in the range ~3600–3000 cm^−1^ (from O-H and N-H groups) and low intensity bands in the range 3000–2700 cm^−1^ (C-H); around 1650 cm^−1^ (COO); 1030–1020 cm^−1^ (C-O), 1080–1070 cm^−1^ (C-H), around 1150 cm^−1^ (C-O-C). Identified bands suggest the dominant presence of polysaccharides in PEs and the presence of polysaccharides and proteins in aqueous extracts. Low intensity bands below 950 cm^−1^ could be due to presence of extracted glucans [1,34]. Mushrooms’ extracts seem to be more diverse in the term of chemical composition. On the other hand, *A. aegerita* and *A. bisporus* extracted polysaccharides ATR-FTIR spectra exhibited more similar bands suggesting similar chemical composition of this fraction. However, ATR-FTIR spectra of *L. sulphureus* extracts differ from other analyzed mushrooms products, indicating different structure of extracted compounds. This observation was confirmed by UHPLC–QToF MS/MS analysis.

The analysis of six mushroom extracts from three edible mushrooms, *A. aegerita, A. bisporus* and *L. sulphureus*, shows that the aqueous extract of *A. aegerita* (AE) contains the highest amount of total and β-glucans and the highest amount of phenolic compounds compared two other analyzed extracts. The proteins are present in all analyzed extracts according to ATR-FTIR spectroscopy, but visible bands on SDS-PAGE gels are observed in *A. aegerita* and *A. bisporus* AE with specific bands as unique markers for their authentication and bioactivities.

### 2.7. Antioxidant Activity

The highest radical-scavenging activity and ferrous-chelating capacity showed aqueous extract of *A. aegerita.* Although *L. sulphureus* exerted the best FRP, other antioxidant activities were significantly lower compared to AE of *A. aegerita* (Table 5). Obtained results indicate that AE of *A. aegerita* has the best antioxidant properties among analyzed samples. Overall, our results showed that AE, which has more identified phenolic compounds than PE, also have better antioxidant potential. These are in accordance with previously published results regarding antioxidant potential of the tested mushrooms, though some of the assays differ [7,13]. According to our knowledge, this is the first study to compare the antioxidant capacities of the polysaccharidic and aqueous extracts of the species *A. bisporus*, *A. aegerita* and *L. sulphureus* from Serbia by acknowledged assays.

### 2.8. Cytotoxicity of Extracts

The results regarding the cytotoxic activity of the mushroom extracts towards HaCaT cells are presented in Table 6. According to the obtained results, the cytotoxicity of different extracts towards HaCaT cell line indicated that only *L. sulphureus* and *A. bisporus* AE were moderately cytotoxic at the concentration of 274.72 and 246.24 µg/mL, respectively. The *A. aegerita* sample did not show this type of activity, since the IC_50_ was over 400 µg/mL. Taofique et al. [35] reported that there is a growing number of natural bioactive substances from mushrooms that can be utilized as cosmeceutical components. Thus, ethanolic extracts from *Pleurotus ostreatus* and *Ganoderma lucidum* were evaluated for their cytotoxic properties on HaCaT and HFF-1 cell lines, as this in vitro assay is the main prerequisite for the safe application of extracts in cosmetic formulations. Our study is the first to point out comparative results regarding the cytotoxicity of selected mushroom extracts in a model of a keratinocyte cell line. These results indicate that our sample extracts are potentially safe for further development in products with added value.

### 2.9. Wound-Healing Properties of Extracts

**Figure 3:**
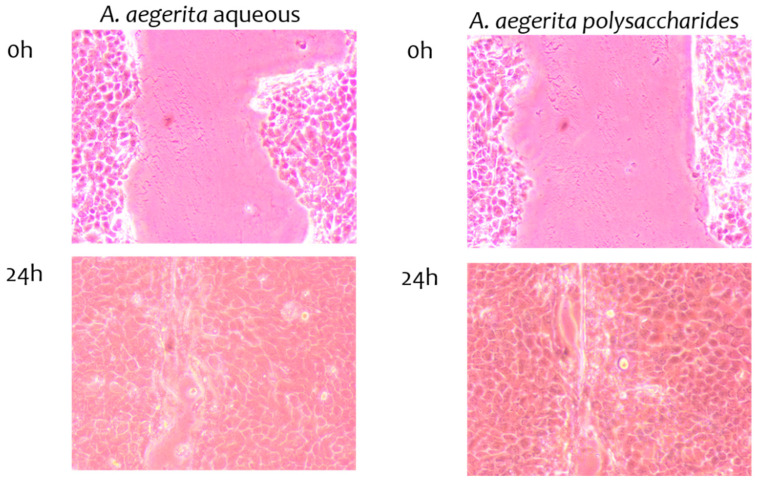
Scratch wound-healing properties of *A. aegerita* extracts.

The results regarding scratch wound-healing properties are presented in Table 7 and Figure 3. The wound-healing assay results indicated that the *A. bisporus* and *L. sulphureus* AEs and PEs promoted wound healing in the HaCaT cell model. The most promising wound-healing activity was observed for the *A. aegerita* PE, which showed complete wound closure after 24 h (Figure 3). This result comes as no surprise, since the extract in question had the highest content of β-glucans (34.95%) among the tested samples, and it showed the best anti-inflammatory potential (described in Section 3.10). As Majtan and Jasenak [36] reported, glucans show promising potential in wound healing due to their pluripotent characteristics. Namely, β-glucans improve wound healing in vivo by promoting macrophage infiltration, which in turn promotes tissue granulation, collagen deposition and re-epithelialization. Herein, we have shown that extracts rich in β-glucans can influence the wound-healing process directly in vitro, probably by different mechanisms that do not involve macrophage infiltration. With their high stability and resistance to wound proteases, β-glucan wound dressings are an excellent product choice for this problem. Given that our results showed that some of the tested extracts had no cytotoxic properties, and in the wound-healing assay they showed quite promising activities, especially in the case of *A. aegerita*, they should be further explored as topical preparations for closing wounds in the pharmaceutical industry. Our study is the first to explore the wound-healing potential of polysaccharidic and aqueous extracts of mushrooms in vitro on a selected model system of skin keratinocytes.

### 2.10. Anti-Inflammatory Properties of Extracts

The results regarding anti-inflammatory activity are presented in Table 8. All mushroom extracts reduced the expression of IL-6 in skin cells. The most promising activity was shown by the tested aqueous and polysaccharide extracts of *A. aegerita*, with IL-6 levels of 4.56 pg/mL (PE) and 4.21 pg/mL (AE). IL-6 production was decreased significantly when HaCaT cells were treated with *S. aureus* as an inflammation-inducing agent and co-treated with mushroom extracts. Our results indicated that the extracts probably interfered with the virulence factors of *S. aureus*, which in turn did not induce IL-6 overexpression in skin cells, pointing to the extract’s infringement on the host–pathogen interaction. According to our knowledge, this is the first study to explore the influence of aqueous and polysaccharidic extracts of mushrooms on IL-6 production in human keratinocytes by using the host–pathogen interactions of the model system (bacterium and human cells).

### 2.11. Antimicrobial Activity Assays

**Figure 4:**
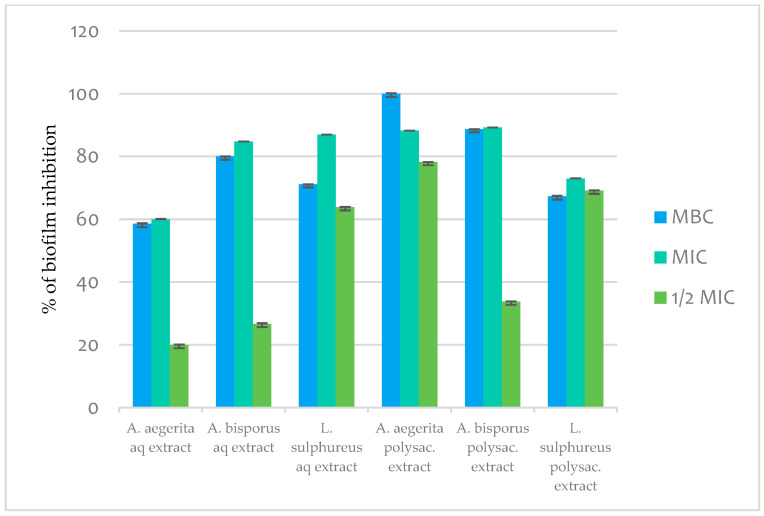
Antibiofilm activity of mushroom aqueous (AEs) and polysaccharide extracts (PEs).

The results regarding antimicrobial activity showed that all the tested extracts had the ability to inhibit the growth of pathogenic microorganisms, including clinical, milk and dairy isolates of bacteria, pathogenic microfungi and yeasts (Table 9 and Table 10, and Figure 4).

#### 2.11.1. Antibacterial Activity

The results regarding antibacterial activity (Table 9) indicated that among the tested bacteria, the *B. cereus* strain was the most susceptible to the activity of the extracts, with an MIC value of 1.00 mg/mL and an MBC of 2.00 mg/mL. This inhibition was achieved with the *A. aegerita* AE, whereas the other extracts exerted lower and rather uniform antibacterial potential towards all the tested strains (with an MIC of 2.00 mg/mL and an MBC of 8.00 mg/mL). The antibacterial activity of the selected extracts was weaker than that of the positive controls; nevertheless, the difference between their activity and tested commercial preservatives does not surpass the fact that the latter are compounds of natural origin, with fewer side effects and being more eco-friendly. Overall, both the AE and PE showed moderate to good activity, especially towards dairy bacterial isolates, which may have practical importance in food technology, given that the aqueous and polysaccharide extracts of these three edible mushrooms could be incorporated into dairy products so as to fortify their functional, organoleptic and sensorial properties. Furthermore, given their noteworthy antioxidant and antimicrobial properties, they could serve as natural preservatives as well. The best activity was demonstrated towards *Y. enterocolitica* with an MIC in the range of 0.25–0.50 mg/mL and an MBC in the range of 0.50–1.00 mg/mL, as well as *L. monocytogenes* dairy strains ATCC 13932, 15313 19111 and 35152, with an MIC in the range of 0.50 mg/mL and an MBC in the range of 1.00–2.00 mg/mL. The lowest activity was observed for the *A. bisporus* AE towards *E. cloacae* (MIC 8.00 mg/mL, MBC 18.00 mg/mL).

**Table 9:** Antibacterial activity of *A. aegerita*, *A. bisporus* and *L. sulphureus* AE and PE (mg/mL).

Microorganisms		*A. aegerita* AE	*A. bisporus* AE	*L. sulphureus* AE	*A. aegerita* PE	*A. bisporus* PE	*L. sulphureus* PE	E211	E224
*S. aureus* (ATCC 11632)	MIC	4.00 c	4.00 c	4.00 c	4.00 c	2.00 b	4.00 c	4.00 c	1.00 a
	MBC	8.00 c	8.00 c	8.00 c	8.00 c	4.00 b	8.00 c	4.00 b	1.00 a
*B. cereus* (clinical isolate)	MIC	1.00 b	2.00 c	2.00 c	4.00 d	2.00 b	4.00 c	0.50 a	2.00 b
	MBC	2.00 b	4.00 c	4.00 c	8.00 d	4.00 c	8.00 d	0.50 a	4.00 c
*L. monocytogenes* (NCTC 7973)	MIC	4.00 d	4.00 d	2.00 c	4.00 d	2.00 c	4.00 d	1.00 b	0.50 a
	MBC	8.00 d	8.00 d	4.00 c	8.00 d	4.00 c	8.00 d	2.00 b	1.00 a
*L. monocytogenes* (ATCC 13932)	MIC	1.00 b	1.00 b	1.00 b	1.00 b	1.00 b	1.00 b	0.50 a	0.50 a
	MBC	2.00 b	2.00 b	2.00 b	2.00 b	2.00 b	2.00 b	1.00 a	1.00 a
*L. monocytogenes* (ATCC 15313)	MIC	0.50 a	0.50 a	0.50 a	0.50 a	1.00 b	0.50 a	0.50 a	1.00 b
	MBC	1.00 a	1.00 a	1.00 a	1.00 a	2.00 b	1.00 a	1.00 a	2.00 b
*L. monocytogenes* (ATCC 19111)	MIC	1.00 a	1.00 a	1.00 a	1.00 a	1.00 a	1.00 a	1.00 a	1.00 a
	MBC	2.00 a	2.00 a	2.00 a	2.00 a	2.00 a	2.00 a	2.00 a	2.00 a
*L. monocytogenes* (ATCC 35152)	MIC	1.00 b	1.00 b	1.00 b	1.00 b	1.00 b	1.00 b	0.50 a	1.00 b
	MBC	2.00 b	2.00 b	2.00 b	2.00 b	2.00 b	2.00 b	1.00 a	2.00 b
*Y. enterocolitica* (ATCC 23715)	MIC	0.50 b	0.25 a	0.50 b	0.50 b	0.50 b	0.50 b	0.50 b	0.50 b
	MBC	1.00 b	0.50 a	1.00 b	1.00 b	1.00 b	1.00 b	1.00 b	1.00 b
*Y. enterocolitica* (ATCC 9610)	MIC	0.50 b	0.50 b	0.25 a	0.50 b	0.25 a	0.25 a	1.00 c	0.50 b
	MBC	1.00 b	1.00 b	0.50 a	1.00 b	0.50 a	1.00 b	2.00 c	1.00 b
*E. coli* (ATCC 25922)	MIC	4.00 d	4.00 d	2.00 c	4.00 d	2.00 c	4.00 d	1.00 b	0.50 a
	MBC	8.00 d	8.00 d	4.00 c	8.00 d	4.00 c	8.00 d	2.00 b	1.00 a
*E. coli* (ATCC 11775)	MIC	1.00 b	1.00 b	1.00 b	1.00 b	1.00 b	1.00 b	0.50 a	0.50 a
	MBC	2.00 b	2.00 b	2.00 b	2.00 b	2.00 b	2.00 b	1.00 a	1.00 a
*E. coli* O157:H (ATCC 700728)	MIC	1.00 b	1.00 b	1.00 b	1.00 b	1.00 b	1.00 b	1.00 b	0.50 a
	MBC	2.00 b	2.00 b	2.00 b	2.00 b	2.00 b	2.00 b	2.00 b	1.00 a
*E. coli* O157:H (ATCC 43888)	MIC	1.00 b	1.00 b	1.00 b	1.00 b	1.00 b	1.00 b	0.50 a	1.00 b
	MBC	2.00 b	2.00 b	2.00 b	2.00 b	2.00 b	2.00 b	1.00 a	2.00 b
*S.* Typhimurium (ATCC 13311)	MIC	4.00 c	4.00 c	2.00 b	4.00 c	2.00 b	4.00 c	1.00 a	1.00 a
	MBC	8.00 d	8.00 d	4.00 c	8.00 d	4.00 c	8.00 d	2.00 b	1.00 a
*E. cloacae* (ATCC 35030)	MIC	4.00 c	8.00 d	2.00 b	4.00 c	2.00 b	4.00 c	2.00 b	0.50 a
	MBC	8.00 c	8.00 c	4.00 b	8.00 c	4.00 b	8.00 c	4.00 b	0.50 a

In each row, different letters mean significant differences between the samples for *p* < 0.05.

#### 2.11.2. Antifungal Activity of Extracts

The results regarding antifungal activity showed a lower inhibitory potential of the tested mushroom extract towards the tested pathogenic fungi than towards the bacteria, with MIC values in the range of 0.5–2.0 mg/mL and MFC values in range of 2.0–8.00 mg/mL (Table 10). In some cases, the tested extracts did not inhibit the growth of micromycetes in the tested concentrations at all, which can be especially observed in the case of *Penicillium verrucosum* var. *cyclopium* (food isolate). The obtained values are comparable to the ones obtained with the positive controls (E221 and E224), which suggests that mushroom extracts may have potential in antifungal drug discovery after all. The most susceptible species to the activity of extracts was *A. versicolor* (Table 10). The results regarding the antifungal activity showed lower potential towards tested micromycetes, with *P. verrucosum* var. *cyclopium* being the most resilient to the activity of the extracts (MFC > 8.00 mg/mL, and *A. fumigatus* being the most susceptible strain (MIC/MFC 0.50/1.00 mg/mL, respectively, in the case of the *L. sulphureus* AE and PE, as well as the *A. aegerita* PE).

**Table 10:** Antifungal activity of *A. aegerita*, *A. bisporus* and *L. sulphureus* AE and PE (mg/mL).

		*A. fumigatus* (ATCC 9197)	*A. versicolor* (ATCC 11730)	*A. niger* (ATCC 6275)	*P. funiculosum* (ATCC 36839)	*P. verrucosum* var. *cyclopium* (food isolate)	*T. viride* (IAM 5061)
*A. aegerita* AE	MIC	2.00 c	2.00 c	1.00 a	2.00 c	2.00 b	1.00 b
	MFC	4.00	4.00 c	2.00 b	4.00 c	4.00 b	2.00 b
*A. bisporus* AE	MIC	1.00 b	2.00 c	1.00 a	2.00 c	2.00 b	1.00 b
	MFC	2.00 b	4.00 c	2.00 b	4.00 c	4.00 b	2.00 b
*L. sulphureus* AE	MIC	0.50 a	1.00 b	1.00 a	>8.00 e	>8.00 c	1.00 b
	MFC	1.00 a	2.00 b	2.00 b	>8.00 e	>8.00 c	2.00 b
*A. aegerita* PE	MIC	0.50 a	0.50 a	1.00 a	4.00 d	>8.00 c	1.00 b
	MFC	1.00 a	1.00 a	2.00 b	8.00 d	>8.00 c	2.00 b
*A. bisporus* PE	MIC	>8.00 c	2.0 0c	>8.00 b	4.00 d	>8.00 c	1.00 b
	MFC	>8.00 c	2.00 b	>8.00 c	8.00 d	>8.00 c	2.00 b
*L. sulphureus* PE	MIC	0.50 a	0.50 a	1.00 a	4.00 d	>8.00 c	1.00 b
	MFC	1.00 a	1.00 a	2.00 b	8.00 d	>8.00 c	2.00 b
E211	MIC	1.00 b	2.00 c	1.00 a	1.00 b	2.00 b	1.00 b
	MFC	2.00 b	4.00 c	2.00 b	2.00 b	4.00 b	2.00 b
E224	MIC	1.00 b	1.00 b	1.00 a	0.50 a	1.00 a	0.50 a
	MFC	1.00 a	1.00 a	1.00 a	0.50 a	1.00 a	0.50 a

In each column, different letters mean significant differences between the samples for *p* < 0.05.

Given the fact that in recent years *Candida* spp. has caused an increasing number of infections in immunocompromised patients, it is of great interest to evaluate naturally sourced substances as potential antifungal agents that are safe for human use. The obtained results regarding anticandidal activity (MICs and MFCs in the range of 0.50–8.0 mg/mL) indicate rather uniform activity regardless of the origin of the tested strain (reference or clinical), as well as a ~100-fold lower anticandidal effect than the commercial antifungal agent ketoconazole (Table 11). However, this does not diminish their potential in the development of novel, efficient pharmaceuticals with antifungal activity. Regarding anticandidal activity, the extracts showed lower potential to inhibit the growth of the tested *Candida* strains than ketoconazole, but some of them are worth mentioning, such as the *L. sulphureus* AE and PE towards *C. albicans* 475/15 and 14/15. These may be attributed to the presence of different groups of compounds identified in our extracts, namely organic and phenolic acids as well as β-glucans.

Overall, the obtained results of antimicrobial activity are in accordance with previously published studies [7,13] and strongly suggest that these edible mushrooms have the potential as antimicrobial agents in both the pharmaceutical and food industries. In the end, this strategy will support sustainable food processing and production, protecting public health and food safety. In the long run, this strategy will support sustainable food processing and production, protecting public health and food safety [37].

#### 2.11.3. Antibiofilm Activity 

The evaluated mushroom extracts could efficiently eradicate 24 h old *S. aureus* biofilms. When a concentration equal to the MBC was applied, the *A. aegerita* PE was able to eradicate 100% of preformed biofilm, while the other agents could induce more than a 50% reduction in biofilm biomass (Figure 4). Microbial biofilms are associated with a range of human diseases, including cystic fibrosis and chronic wounds, and due to their antibiotic resistance are responsible for the prolonged duration of antibiotic treatment and chronic infections. Bearing in mind the urgent need for the discovery of an efficient antibiofilm approach, the recorded activities of extracts (Figure 4) are of great interest and at the same time are the first evidence of antibiofilm activities. Our results are in accordance with those of Moussa et al. [38], which suggest that certain compounds identified in mushrooms may be regarded as potent antibiofilm agents in *S. aureus* (the result we obtained in this study as well), *Enterococcus faecium*, *Klebsiella pneumoniae*, *Acinetobacter baumannii*, *Pseudomonas aeruginosa*, etc.

## 3. Material and Methods

### 3.1. Standards and Reagents

Solvents for analyses (acetonitrile and formic acid) were of LC–MS grade (Fisher Scientific, Loughborough, UK). Methanol and ethanol (HPLC grade) were purchased from AppliChem (Cheshire, CT, USA). Ultrapure water was generated by deionization (Millipore, Billerica, MA, USA) system. Analytical standards as well as other chemicals were purchased from Sigma-Aldrich (Steinheim, Germany) unless stated otherwise.

### 3.2. Mushroom Collection and Identification

Fruiting bodies of wild growing mushrooms *L. sulphureus* and *A. aegerita* were collected in Zvezdara forest, Belgrade in 2021 and determined by Dr. Jasmina Glamočlija, PhD, Institute for Biological Research “Siniša Stanković”, National Institute of Republic of Serbia, University of Belgrade, Serbia. A voucher specimen has been deposited at the Fungal Collection Unit of the Mycological Laboratory, Department for Plant Physiology, Institute for Biological Research “Siniša Stanković”, Belgrade, Serbia. The samples were freeze-dried (LH Leybold, Lyovac GT2, Frankendorf, Switzerland), reduced to a fine dried powder (20 mesh), mixed to obtain homogenous samples and stored at 4 °C, protected from light, until further analysis. Fruiting bodies of commercially cultivated *A. bisporus* were kindly provided by Ekofungi doo, Zrenjaninski put bb, Palilula, Beograd 11213. 

### 3.3. Preparation of Mushroom Extracts

Aqueous extracts (AEs) were prepared according to the procedure previously published by Vamanu and Nita [39] with some modifications. Dried mushroom sample (10 g) was extracted with 200 mL of MilliQ water for 90 min in an ultrasonic bath at room temperature. After extraction, the extract was filtered (Whatman No. 4) and the residue was re-extracted two more times following the same procedure. Combined extracts were lyophilized and stored in a freezer until further use. 

Polysaccharide extract (PE) was prepared using a modified method from Cheng et al. [40] using the following procedure: dried mushroom sample (10 g) was extracted with 300 mL of 96% ethanol for 120 min at room temperature with a magnetic stirrer; subsequently, the suspension was centrifuged for 20 min at 1200 rpm. Residual supernatant was discarded, while the pellets were suspended in 300 mL of MilliQ water and cooled down for 20 min at 4 °C. The suspension was then autoclaved at 120 °C for 20 min (Raypa, Barcelona, Spain), after which it was cooled on ice. The mixture was then centrifuged at 8000 rpm for 20 min, and the clear supernatant was further used. Two volumes of 96% ethanol were added, and the mixture was left overnight at 4 °C on a magnetic stirrer, after which the polysaccharides were collected by precipitation. The pellets, dissolved in 20 mL of 0.02 M Tris buffer, pH 7.4, were collected after centrifugation for 15 min at 5000 rpm. To remove unwanted compounds, dissolved pellets were dialyzed overnight at 4 °C using the same Tris buffer. The resulting sample was then centrifuged at 8000 rpm for 15 min, and two volumes of 96% ethanol were added to the supernatant for re-precipitation. The sample was stored at 4 °C for 30 min, after which it was centrifuged at 8000 rpm for 20 min. Ethanol was discarded and evaporated, and the remaining pellets were dissolved in water, lyophilized and stored in a freezer until further use.

The preparation of AE and PE was repeated three times to obtain representative samples for further analysis.

### 3.4. Total α-Glucan and β-Glucan Content

Determination of total and α-glucan content in lyophilized polysaccharide and aqueous extracts of *A. bisporus, L. sulphureus* and *A. aegerita* was performed using Mushroom and Yeast β- glucan Assay K-YBGL 12/2024 (Megazyme^©^ International Ireland Ltd., Wicklow, Ireland, 2021), according to manufacture specification. The values were calculated using Mega-Calc^TM^ available online and expressed in percentages. The experiment was performed in triplicate.

### 3.5. Total Protein Content

The determination of soluble protein content in aqueous extracts of *A. bisporus, L. sulphureus* and *A. aegerita* was performed using the Bradford method [41], using bovine serum albumin (BSA) as the standard. The absorbance of all solutions was measured spectrophotometrically at 517 nm. Protein concentrations are expressed as g of BSA equivalents per 100 g. The experiment was performed in triplicate.

### 3.6. Total Phenolic Content

The total phenolic content (TPC) was determined using Folin–Ciocalteu’s reagent as previously described by Milinčić et al. [42,43]. Briefly, an aliquot of sample (70 µL) was mixed with 300 µL of Folin–Ciocalteu’s reagent and 230 µL of 7.5% Na_2_CO_3_, followed by incubation for 90 min at room temperature. The absorbance of all solutions was measured spectrophotometrically at 765 nm. The results are expressed as g of gallic acid equivalents per 100 g of the sample (g GAE/100 g). The experiment was performed in triplicate.

### 3.7. Protein Profile of Mushroom Aqueous Extracts

SDS-PAGE in reducing conditions was performed according to the procedure previously published by Pesic et al. [44] in the following manner: separating gels (12.5% *w*/*v*; pH = 8.85) and stacking gels (5% *w*/*v*; pH = 6.8), with Tris-glycine as a running buffer (0.05 M Tris pH = 8.5), and 0.19 M glycine, 0.1% *w*/*v* SDS). Samples (50 mg) were dissolved in 1 mL of buffer containing 0.055 M Tris-HCl (pH = 6.8), 2% (*w*/*v*) SDS, 7% (*v*/*v*) glycerol, 0.0025% (*w*/*v*) bromophenol blue and 5% β-mercaptoethanol. Aliquots of samples (50 µL) were loaded into wells, after which electrophoresis was performed under the following conditions: Current 30A for the first 30 min, then 60A while the voltage was set to a maximum of 500 V. Electrophoresis was carried out for 3 h. After separation, gels were stained using Coomassie blue dye solution [0.23 g/100 g Coomassie^®^ brilliant blue R250, 3.9 g/100 g trichloroacetic acid, 6 mL/100 mL acetic acid and 17 mL/100 mL methanol] for 45 min, then destained, scanned and analyzed using SigmaGel software (GelAnalyzer 19.1 software). The molecular weight of detected polypeptide bands were evaluated using the molecular weight standard as previously described by Pesic et al. [44]. The experiment was performed in triplicate.

### 3.8. Phenolic Profile of Mushroom Aqueous Extracts

#### 3.8.1. Preparation of Samples

Lyophilized aqueous and polysaccharide samples were extracted with 80% methanol containing 0.1% HCl. Samples were vigorously mixed for 60 min at room temperature, after which they were centrifuged at 17,000× *g* for 5 min and filtered through 0.45 µm filters. Collected supernatants were used for further chromatographic analysis.

#### 3.8.2. UHPLC–QToF-MS Analysis

The analyses (characterization and identification) of bioactive compounds were carried out on Agilent 1290 Infinity ultra high-performance liquid chromatography (UHPLC) system coupled with a quadrupole time-of-flight mass spectrometry (6530C QToF-MS) from Agilent Technologies, Inc., Santa Clara, CA, USA. The chromatographic separation was performed according to the procedure described by Milinčić et al. [45] at 40 °C on a Zorbax C18 column (2.1 × 50 mm, 1.8 µm) from Agilent Technologies, Inc., Santa Clara, CA, USA. The mobile phase mixtures comprised (A) ultrapure water and (B) acetonitrile (MS grade), with both A and B containing 0.1% formic acid. The flow rate was set to 0.3 mL min^−1^, and the injection volume was 5 µL. The gradient elution program started with 2% solvent B for the first 2 min, which then reached 98% B over the next 17 min, and for the next 5 min the gradient was returned to initial conditions (2% B) to re-equilibrate the column. The QToF-MS system was equipped with an Agilent Jet Stream electrospray ionization (ESI) source, operating in both positive (ESI+) and negative (ESI-) ionization modes. All identified compounds (organic acid, phenolic acid and other derivatives) were confirmed in negative ionization mode, using Auto MS/MS acquisition (*m*/*z* = 100–1500, scan rate 5 spectra/s) and collision energy set at 30 eV. The operation parameters for the ESI were set as follows: nebulizer pressure of 45 psi, drying gas temperature of 225 °C and flow rate of 8 L/min, sheath gas temperature of 300 °C and sheath gas flow rate of 10 L/min, capillary voltage of 2500 V, fragmentor energy of 175 V, skimmer voltage of 65 V and octopole RF peak at 750 V. The QToF-MS system recorded spectra over the *m*/*z* range of 100–1500 with a scan rate of 2 Hz. Agilent Mass Hunter software (Mass Hunter workstation for LC/QToFM5960AA) was used for data evaluation and analysis. Organic acids and phenolics were identified based on *m*/*z* exact mass, typical MS fragments and available literature data. The exact masses of the compounds were calculated using ChemDraw software (version 12.0, CambridgeSoft, Cambridge, MA, USA).

### 3.9. ATR-FTIR Spectroscopy

The obtained extracts were analyzed using an IRAffinity-1 spectrometer equipped with an ATR unit (Shimadzu, Kyoto, Japan). The spectra were collected in the wavenumber range of 4000–600 cm^−1^, at a resolution of 4 cm^−1^, from 100 scan accumulations.

### 3.10. Antioxidant Assay

#### 3.10.1. FRAP Assay

The ferric-reducing power assay was conducted according to the method previously described by Pešić et al. [46]. Briefly, an aliquot of diluted sample (250 μL) was mixed with 0.2 M phosphate buffer, pH 6.6 (250 μL), and 1% potassium ferricyanide solution (250 μL), after which the mixture was incubated for 20 min at 50 °C. Subsequently, 10% TCA (250 μL) was added, and the resulting mixture was centrifuged at 17,000× *g* (Sigma 201M Centrifuge, Osterode am Harz, Germany) for 5 min. The resulting supernatant (500 μL) was combined with MilliQ water (500 μL) and 0.1% ferric chloride (100 μL) and incubated for 10 min, after which the absorbance was measured at 700 nm (UV-1800, Shimadzu USA Manufacturing Inc., Canby, OR, USA). The experiment was performed in triplicate and the results are expressed as µg of ascorbic acid equivalents per mL of sample (µg AA/mL).

#### 3.10.2. ABTS•^+^ Assay

ABTS^+^ free-radical-scavenging activity was determined according to the procedure described by Milinčić et al. [43]. The stock solution (7 mM aqueous solution of ABTS 2,2-azino-bis/3-ethil-benothiazoline-6-sulphonic acid with 2.45 mM potassium persulfate) was incubated in the dark for 16 h. The working solution of ABTS•^+^ was prepared by diluting the stock solution with methanol to obtain an absorbance between 0.7 and 0.8 at 734 nm. Thereafter, 10 μL of sample was mixed with 1 mL of ABTS•^+^ working solution, and after 7 min the absorbance was measured at 734 nm (UV-1800, Shimadzu USA Manufacturing Inc., Canby, OR, USA). Values were calculated according to the equation described by Pesic et al. [46]. The experiment was performed in triplicate and the results are expressed as µg of ascorbic acid equivalents per mL of sample (µg AA/mL).

#### 3.10.3. DPPH• Assay

The DPPH^•^ assay was performed according to the procedure previously published by Milinčić et al. [43]. Briefly, samples (105 μL) were mixed with DPPH^•^ working solution (840 μL) and incubated in the dark for 30 min, after which the absorbance was measured at 515 nm (UV-1800, Shimadzu USA Manufacturing Inc., Canby, OR, USA). The values are expressed as μg/mL of Trolox equivalents per mL of sample (µg TE/mL). The experiment was performed in triplicate.

### 3.11. Cytotoxicity towards HaCaT Cells

The cytotoxic effect of extracts was determined on a spontaneously immortalized keratinocyte cell line (HaCaT) using the crystal violet assay as described previously by Stojković et al. [47]. The samples were dissolved in PBS to a final concentration of 8 mg/mL. HaCaT cells were grown in high-glucose Dulbecco’s Modified Eagle Medium (DMEM) supplemented with 10% fetal bovine serum (FBS), 2 mM L-glutamine and 1% antibiotic–antimycotic (Invitrogen) at 37 °C in a 5% CO_2_ incubator. Cells (10^4^ cells/well) were seeded in a 96-well microtiter plate with an adhesive bottom. After 48 h, the medium was removed and the cells were treated with various concentrations of the extracts in triplicate wells during the 24 h. Afterwards, the medium was removed and the cells were washed twice with phosphate-buffered saline (PBS) and stained with 0.4% crystal violet staining solution for 20 min. Crystal violet staining solution was removed and cells were washed in a stream of tap water and left to air dry at room temperature. The absorbance of dye dissolved in methanol was measured at 570 nm (OD_570_) in a plate reader. The results are expressed as IC_50_ values, indicating 50% of cell viability when compared with untreated controls. The solvent was used as a negative control. The following criteria were used to categorize the cytotoxic activity of extracts to the HaCaT cell line: IC_50_ ≤ 20 μg/mL = highly cytotoxic, IC_50_ ranging between 21 and 250 μg/mL = moderately cytotoxic, IC_50_ ranging between 201 and 400 μg/mL = weakly cytotoxic, and IC_50_ > 401 μg/mL = no cytotoxicity.

### 3.12. Scratch Wound-Healing Assay

The assay was performed as described by Stojković et al. [48]. HaCaT cells were grown until reaching confluence. The cell monolayer was scratched with a sterile 200 μL tip. Floating cells were washed, and cells were incubated in reduced DMEM supplemented with 1% FBS, 2 mM L-glutamine and 1% antibiotic–antimycotic (Invitrogen), and containing extracts that were used in the IC_25_ concentration determination in the cytotoxicity assay. Cell migration was monitored with Nikon Eclipse TS2 (Amsterdam, Netherland) 24 h after the wound was made and treated. An untreated control was used to measure wound closure under these conditions, without the addition of extracts. The experiment was performed in triplicate and the results are presented as percentages of wound closure during the exposure to the tested extracts. 

### 3.13. Anti-Inflammatory Properties 

Anti-inflammatory properties were measured as the modulation of interleukin-6 (IL-6) levels in HaCaT cells in response to bacteria and extracts. For this assay, the confluent monolayer of HaCaT cells was grown in 6-well plates with an adhesive bottom. Medium was removed and fresh FBS-free DMEM with the tested compounds was added, after which the plate was incubated at 37 °C for 15 min in a 5% CO_2_ incubator. Subsequently, *S. aureus* culture (10^8^ CFU/mL, 100 µL) was added to the wells, and the mixture was incubated at 37 °C for 4 h. The supernatant was collected, centrifuged at 1000 rpm for 10 min and used for the determination of interleukin-6 and interleukin-8 levels using the Human IL-6 ELISA Kit (Invitrogen, Vienna, Austria) as described by the manufacturer. The level of IL-6 was determined in untreated HaCaT cells, HaCaT cells inoculated with *S. aureus* and HaCaT cells treated with extracts and inoculated. The experiment was performed in triplicate.

### 3.14. Antimicrobial Activity Assay

#### 3.14.1. Microorganisms

The following Gram (+) bacteria were tested: *Staphylococcus aureus* (ATCC 11632), *Bacillus cereus* (clinical isolate) and *Listeria monocytogenes* (NCTC 7973), *L. monocytogenes* (ATCC 13932), *L. monocytogenes* (ATCC 15313), *L. monocytogenes* (ATCC 19111) and *L. monocytogenes* (ATCC 35152), as well as the following Gram (−) bacteria: *Yersinia enterocolitica* (ATCC 23715), *Y. enterocolitica* (ATCC 9610), *Escherichia coli* (ATCC 25922), *E. coli* (ATCC 11775), *E. coli* O157:H7 (ATCC 700728), *E. coli* O157:H7 (ATCC 43888), *Enterobacter cloacae* (ATCC 35030) and *Salmonella* Typhimurium (ATCC 13311); as for the tested micromycetes, the following were used: *Aspergillus fumigatus* (ATCC 9197), *Aspergillus niger* (ATCC 6275), *Aspergillus versicolor* (ATCC 11730), *Penicillium funiculosum* (ATCC 36839), *Penicillium verrucosum* var. *cyclopium* (food isolate) and *Trichoderma viride* (IAM 5081). The microorganisms were deposited at the mycological laboratory of the Department of Plant Physiology, Institute for Biological research “Sinisa Stanković”, National Institute of Republic of Serbia, University of Belgrade, Serbia. Regarding anticandidal activity, the following strains were evaluated: *Candida albicans* 475/15, *C. albicans* 13/15, *C. albicans* 17/15, *C. parapsilosis* ATCC 22019, *C. tropicalis*, ATCC 750 and *C. krusei* H1/16.

#### 3.14.2. Antibacterial Activity and Anticandidal Activity

The antibacterial assay was carried out by a modified microdilution method [49]. Bacterial strains were cultured overnight at 37 °C in Tryptic Soy Broth, after which they were adjusted with sterile saline to a concentration of 1.0 × 10^5^ CFU/mL. Samples dissolved in 30% ethanol were added to Tryptic Soy Broth (TSB) medium (100 µL) with bacterial inoculum (1.0 × 10^4^ CFU per well). After incubation (24 h at 37 °C), *p*-iodonitrotetrazolium chloride (40 μL, 0.2 mgmL^−1^) was added to each well of the plate and further incubated for 60 min at 37 °C for color development. The lowest concentrations that showed a distinct reduction in color intensity—light red in comparison to the intensive red in the control well (with no added compounds), or an absence of color—were defined as the minimal inhibitory concentrations (MICs). The minimal bactericidal concentrations (MBCs) were determined by serial subcultivation of 2 µL into the wells already containing 100 µL of broth and further incubation for 24 h at 37 °C. The lowest concentration with no visible growth was defined as the MBC, indicating 99.5% killing of the original inoculum. E211 and E224 were used as positive controls for bacteria, and ketoconazole for *Candida* spp., and the experiments were performed in triplicate.

#### 3.14.3. Antifungal Activity

Prior to the microdilution method, fungal spores were washed from the surface of agar plates with sterile 0.85% saline containing 0.1% Tween 80 (*v*/*v*). Samples dissolved in 30% DMSO were added to broth Malt medium, after which fungal inoculum was added. Plates were incubated at 25 °C for 5 days. The lowest concentrations with significant reduction in mycelial growth (at the binocular microscope) were defined as the MICs. The minimal fungicidal concentrations (MFCs) were determined by serial subcultivation of 2 µL of the tested sample dissolved in medium and further incubated for 72 h at 25 °C. The lowest concentration with no visible growth was defined as the MFC, indicating 99.5% killing of the original inoculum. The experiment was performed in triplicate.

#### 3.14.4. Inhibition of Biofilm Formation

The ability of mushroom extracts to inhibit biofilm formation was determined as described previously, with some modifications [50]. *S. aureus* cells were incubated in 96-well microtiter plates with an adhesive bottom (Sarstedt, Germany) with MBC, MIC and sub-MIC concentrations of the tested extracts at 37 °C for 24 h. Then, wells were washed twice with sterile PBS, pH 7.4, and biofilms were fixed with methanol for 10 min; next, methanol was removed, and the plate was air-dried and stained with 0.1% crystal violet (Bio-Merieux, France) for 30 min. Wells were washed with water, air-dried, and 100 μL of 96% ethanol (Zorka Pharma, Šabac, Serbia) was added to dissolve the bounded crystal violet. The absorbance was read at 570 nm using a microplate reader Multiskan™ FC Microplate Photometer, Thermo Scientific™. The percentage of inhibition of biofilm formation was calculated by the following formula: Inhibition (%) = [(A_control_ − A_sample_)/A_control_] × 100. 

### 3.15. Statistical Analysis

The experiments were performed in triplicate, and the results are presented as mean ± standard error. The data were statistically analyzed using GraphPad PRISM 8.0.2 software. The differences between the control and experimental groups were compared using the multiple *t*-test, one way ANOVA and two-way ANOVA. A *p*-value < 0.05 was considered to be statistically significant.

## 4. Conclusions

The study demonstrated that polysaccharide extracts (PEs) from mushrooms generally contain higher total and β-glucan levels compared to aqueous extracts (AEs). Notably, *A. aegerita* exhibited the highest content of total and β-glucans, highlighting its potential as a significant source of these bioactive compounds. *L. sulphureus* stood out with a significantly higher total protein content compared to *A. aegerita* and *A. bisporus*, indicating its potential as a valuable protein source. This aligns with the broader understanding of mushrooms as rich protein sources with complete amino acid profiles. The highest phenolic content was found in the AE of *A. aegerita*, which also exhibited superior antioxidant properties. This reinforces the importance of phenolic compounds in contributing to the bioactivity of mushroom extracts.

SDS-PAGE analysis revealed distinct polypeptide profiles for the *A. aegerita* and *A. bisporus* AEs, suggesting unique bioactivities and potential for authentication based on specific protein markers. UHPLC–QToF-MS analysis highlighted a diverse range of organic and phenolic acids in the extracts, with the *A. aegerita* AE showing the highest number of detected compounds. These profiles contribute to the understanding of the antioxidant and biological properties of the extracts. ATR-FTIR analysis confirmed the dominant presence of polysaccharides in the PEs and a mix of polysaccharides and proteins in the AEs. The chemical composition of extracts from different mushrooms varied, suggesting different bioactive potentials.

The *A. aegerita* AE demonstrated the highest antioxidant activity, suggesting it as the most potent extract among those analyzed. This is consistent with its high phenolic content. The study found that the *L. sulphureus* and *A. bisporus* AEs exhibited moderate cytotoxicity towards HaCaT cells, while *A. aegerita* showed no significant cytotoxicity, indicating its safety for further development in cosmetic applications. The *A. aegerita* PE showed the most promising wound-healing activity, completely closing wounds in vitro within 24 h. This highlights its potential for developing topical wound-healing products. All mushroom extracts reduced IL-6 expression in skin cells, with the *A. aegerita* extracts showing the most significant reduction, pointing to their potential in managing inflammation.

The extracts displayed notable antimicrobial properties, particularly against pathogenic bacteria and fungi. The *A. aegerita* AE was particularly effective against *B. cereus*, indicating its potential as a natural antimicrobial agent in food and pharmaceutical industries. The mushroom extracts, especially the *A. aegerita* PE, effectively eradicated preformed biofilms of *S. aureus*, suggesting their potential as antibiofilm agents, which is crucial for combating antibiotic-resistant infections. Overall, the study underscores the significant bioactive potential of mushroom extracts, particularly those from *A. aegerita*, highlighting their application in therapeutic, cosmetic and food industries.

Furthermore, as we demonstrated that *A. aegerita* had the highest anti-inflammatory potential and activity towards *S. aureus*, which can cause skin infections, we should definitely aim towards the development of products for topical application to wounds, with prolonged exposure to external conditions that can cause skin contamination and infection.

## Figures and Tables

**Table 1:** Total, α- and β-glucan content in lyophilized polysaccharide and aqueous extracts of *A. bisporus, L. sulphureus* and *A. aegerita* expressed as mean ± standard deviation.

	Total Glucan Content (%)	α-Glucans (%)	β-Glucans (%)
Polysaccharide extracts			
*A. aegerita*	44.28 ± 0.06	9.34 ± 0.86	34.95 ± 0.80
*A. bisporus*	33.37 ± 0.31	12.64 ± 1.82	20.73 ± 2.13
*L. sulphureus*	11.47 ± 0.38	0.74 ± 0.03	10.73 ± 0.35
Aqueous extracts			
*A. aegerita*	25.47 ± 2.22	14.36 ± 0.57	11.10 ± 2.79
*A. bisporus*	7.32 ± 0.07	2.54 ± 0.02	4.77 ± 0.09
*L. sulphureus*	6.92 ± 1.45	2.27 ± 0.03	4.65 ± 0.05

**Table 2:** Total protein content in lyophilized aqueous extracts of *A. bisporus, L. sulphureus* and *A. aegerita* expressed as mean ± standard deviation.

	Total Protein Content (g BSA/100 g dw)	
	Aqueous Extracts	Polysaccharide Extracts
*A. aegerita*	1.84 ± 0.02	n.d. *
*A. bisporus*	2.70 ± 0.07	n.d. *
*L. sulphureus*	5.62 ± 0.91	n.d. *

n.d. *—not detected.

**Table 3:** Total phenolic content in lyophilized aqueous extracts of *A. bisporus*, *L. sulphureus* and *A. aegerita* expressed as mean ± standard deviation.

	Total Phenolic Content (g GAE/100 g dw)	
	Aqueous Extracts	Polysaccharide Extracts
*A. aegerita*	23.72 ± 0.02	n.d. *
*A. bisporus*	7.9 ± 0.07	n.d. *
*L. sulphureus*	0.93 ± 0.91	0.19 ± 0.01

n.d. *—not detected.

**Table 4:** Characterization of bioactive compounds of *L. sulphureus*, *A. aegerita* and *A. bisporus* AEs and PEs by UHPLC–QToF-MS. Target compounds, mean expected retention times (RTs), molecular formula, calculated mass, *m*/*z* exact mass, mean mass accuracy (mDa) and MS/MS fragments are presented.

RT, min	Compounds Name	Formula	Calculated Mass	*m*/*z* Exact Mass	mDa	MS^2^ Fragments (% Base Peak)	Samples						Refs
							ABP	ABA	AAP	AAA	LSP	LSA	
***Organic acids and derivatives***													
0.73	Fumaric acid	C_4_H_3_O_4_^–^	115.00368	115.00491	−1.23	**/**	+	+	+	+	−	+	[22,23]
0.73	Malic acid	C_4_H_5_O_5_^–^	133.01425	133.01571	−1.46	**115.0050 (100)**	+	+	+	+	+	+	[22,23]
0.83	Citric acid	C_6_H_7_O_7_^–^	191.01973	191.02299	−3.26	**111.0093 (100)**, 112.01131	+	+	+	+	+	+	[22,23]
0.94	Succinic acid	C_4_H_5_O_4_^–^	117.01933	117.02020	−0.87	**/**	+	+	−	+	+	+	[22]
6.27	Pimelic acid (6-Carboxyhexanoate)	C_7_H_11_O_4_^–^	159.06628	159.06686	−0.58	**115.07504 (100)**, 116.08119, 118.04377	−	+	−	+	+	+	/
7.88	3-hydroxy sebacic acid	C_10_H_17_O_5_^–^	217.10815	217.10986	−1.72	**171.1020 (100)**, 127.1115, 132.04799, 153.06758	−	+	−	−	+	+	[33]
8.49	Azelaic acid	C_9_H_15_O_4_^–^	187.09758	187.09846	−0.88	**123.0813 (100)**, 125.0971	+	+	+	+	+	+	[24]
8.56	Diethyl 3-hydroxyglutarate	C_9_H_15_O_5_^–^	203.09250	203.09342	−0.92	**113.0977 (100)**, 129.0912, 141.0931, 147.0154	−	−	−	+	+	+	/
9.31	Sebacic acid	C_10_H_17_O_4_^–^	201.11323	201.11399	−0.76	**139.1132 (100)**, 111.0815, 137.0974, 183.1025, 184.1039	−	+	+	+	+	+	[24]
10.58	Pinellic acid	C_18_H_33_O_5_^–^	329.23335	329.23534	−2.00	**171.1047 (100)**, 139.1139, 183.1382, 201.1145, 211.1345, 229.1449	+	+	+	+	+	+	[25]
13.41	9-Hydroxy-13-oxo-10-octadecenoic acid	C_18_H_31_O_4_^–^	311.22278	311.22509	−2.31	**201.1138 (100)**, 127.1115, 139.1125, 171.1032, 183.1325, 197.1195	−	+	−	+	+	+	[26]
15.84	13-Hydroxy-9,11-octadecadienoic acid	C_18_H_31_O_3_^–^	295.22787	295.22894	−1.07	**127.1132 (100)**, 111.0818, 171.1024, 183.1032	+	+	+	+	+	+	[26]
***Phenolic acids and derivatives***													
1.28	Gallic acid	C_7_H_5_O_5_^–^	169.01425	169.01871	−4.46	**125.0240 (100)**, 124.0164, 107.0145, 108.0226	−	−	−	−	+	−	[27]
2.49	3,4-Dihydroxybenzoic acid (like Protocatehuic acid)	C_7_H_5_O_4_^–^	153.01933	153.02228	−2.95	**108.0215 (100)**, 109.0295	−	+	−	−	+	+	[27,28]
4.30	*p*-Hydroxybenzoic acid	C_7_H_5_O_3_^–^	137.02390	137.02649	−2.59	**/**	−	+	−	+	+	+	[27,28]
4.31	4-Methoxybenzoic acid (like *p*-Anisic acid)	C_8_H_7_O_3_^–^	151.04007	151.04025	−0.18	**108.0219 (100)**, 107.0389, 109.0327	−	−	−	+	−	+	[29]
4.44	3,4-Dimethoxybenzoate (like veratric acid)	C_9_H_9_O_4_^–^	181.05063	181.05262	−1.98	**134.0381 (100)**, 107.0569, 135.0427, 137.0216, 119.0531	−	−	−	+	+	+	[30]
6.19	Coumaric acid is. I	C_9_H_7_O_3_^–^	163.04007	163.04142	−1.35	**117.0338 (100)**, 118.0352, 119.0237	−	−	−	+	−	+	[27,28]
7.28	*p*-hydroxy-hydrocinnamic acid (or Dihydrocoumaric acid)	C_9_H_9_O_3_^–^	165.05572	165.05574	−0.02	**119.0504 (100)**, 117.0343, 147.0475	−	+	−	+	+	+	/
7.58	Vanillic acid	C_8_H_7_O_4_^–^	167.03498	167.03916	−4.18	**105.0345 (100)**, 123.0457	−	−	−	−	+	+	[30,31]
7.81	Sinapic acid	C_11_H_11_O_5_^–^	223.06120	223.06138	−0.18	**164.0408 (100)**, 117.0675, 133.0661, 163.0411, 179.0703, 193.0171	−	+	−	+	−	−	[31]
9.77	Coumaric acid is. II	C_9_H_7_O_3_^–^	163.04007	163.04023	−0.17	**117.0357 (100)**, 119.0454, 145.9088	−	−	−	+	−	+	[28,30]
11.80	Ethyl-homovanillate	C_11_H_13_O_4_^–^	209.08193	209.08451	−2.58	**122.0374 (100)**, 135.0455, 150.0693	−	−	−	−	+	−	/
***Other compounds***													
13.82	Triterpenoid (like maslinic acid)	C_30_H_47_O_4_^–^	471.34798	471.34992	−1.94	**471.3453 (100)**	−	−	−	−	+	+	[32]

Abbreviations: ABA—*A. bisporus* aqueous extract; ABP—*A. bisporus* polysaccharide extract; LSA—*L. sulphureus* aqueous extract; LSP—*L. sulphureus* polysaccharide extract; AAA—*A. aegerita* aqueous extract; AAP—*A. aegerita* polysaccharide extract. “−“, nonidentified compounds; “+”, identified compounds.

**Table 5:** Antioxidant activity of mushroom extracts expressed as mean ± standard deviation.

Mushroom Extracts	ABTS (g Trolox/100 g)	FRP (µg AA/mL)	FCC (µg EDTA/mL)
*A. aegerita*, aqueous	21.97	43.56	48.25
*A. bisporus*, aqueous	9.07	7.26	47.9
*L. sulphureus*, aqueous	4.92	219.52	0.19
*L. sulphureus*, polysaccharides	3.37	42.05	2.49

**Table 6:** Cytotoxicity of mushroom extracts expressed as mean ± standard deviation.

Mushroom Extracts	IC_50_ Value (µg/mL)
*A. bisporus*, aqueous	246.24 ± 5.46
*L. sulphureus*, aqueous	274.72 ± 2.54
*A. aegerita*, aqueous	>400
*A. bisporus*, polysaccharides	>400
*L. sulphureus*, polysaccharides	>400
*A. aegerita*, polysaccharides	>400

**Table 7:** Scratch wound-healing properties of *A. bisporus* and *L. sulphureus* extracts expressed as mean ± standard deviation.

Mushroom Extracts	Wound Closure %
*A. bisporus*, aqueous	65.96 ± 6.98
*L. sulphureus*, aqueous	62.56 ± 9.43
*A. bisporus*, polysaccharides	84.27 ± 4.54
*L. sulphureus*, polysaccharides	77.06 ± 7.65

**Table 8:** Anti-inflammatory properties of mushroom extracts expressed as mean ± standard deviation.

Mushroom Extracts	IL-6 (pg/mL)
Control	2.96 ± 0.02
*S. aureus*	16.64 ± 0.06
*S. aureus* + AE *A. aegerita*	4.56 ± 0.08
*S. aureus* + PE *A. aegerita*	4.21 ± 0.02
*S. aureus* + AE *A. bisporus*	7.42 ± 0.02
*S. aureus* + PE *A. bisporus*	6.78 ± 0.04
*S. aureus +* AE *L. sulphureus*	15.12 ± 0.10
*S. aureus +* PE *L. sulphureus*	10.21 ± 0.09

**Table 11:** Anticandidal activity of *A. aegerita*, *A. bisporus* and *L. sulphureus* AE and PE (mg/mL).

		*C. albicans* 475/15	*C. albicans* 13/15	*C. albicans* 17/15	*C. parapsilosis* ATCC 22019	*C. tropicalis* ATCC 750	*C. krusei* H1/16
*A. aegerita* AE	MIC	2.00 d	2.00 d	2.00 b	1.00 b	2.00 c	4.00 c
	MFC	4.00 d	4.00 d	4.00 b	2.00 b	4.00 c	8.00 c
*A. bisporus* AE	MIC	1.00 c	2.00 d	2.00 b	1.00 b	2.00 c	4.00 c
	MFC	2.00 c	4.00 d	4.00 b	2.00 b	4.00 c	8.00 c
*L. sulphureus* AE	MIC	0.50 b	0.50 b	4.00 c	1.00 b	1.00 b	4.00 c
	MFC	1.00 b	1.00 b	8.00 c	2.00 b	2.00 b	8.00 c
*A. aegerita* PE	MIC	0.50 b	0.50 b	2.00 b	1.00 b	2.00 c	4.00 c
	MFC	1.00 b	1.00 b	4.00 b	2.00 b	4.00 c	8.00 c
*A. bisporus* PE	MIC	1.00 c	1.00 c	2.00 b	1.00 b	2.00 c	2.00 b
	MFC	2.00 c	2.00 c	4.00 b	2.00 b	4.00 c	4.00 b
*L. sulphureus* PE	MIC	0.50 b	1.00 c	2.00 b	1.00 b	2.00 c	2.00 b
	MFC	1.00 b	2.00 c	4.00 b	2.00 b	4.00 c	4.00 b
Ketoconazole (× 10^−3^)	MIC	3.20 a	1.60 a	1.60 a	3.20 a	1.60 a	1.60 a
	MFC	6.40 a	51.20 a	51.20 a	6.40 a	6.40 a	3.20 a

In each column, different letters mean significant differences between the samples for *p* < 0.05.

## Data Availability

The original contributions presented in the study are included in the article/Supplementary Material, further inquiries can be directed to the corresponding authors.

## Supplementary Materials

The following supporting information can be downloaded at: https://www.mdpi.com/article/10.3390/ph17091153/s1, Table S1. Polypeptide composition (%) of mushroom aqueous extract of *A. bisporus* and *A. aegerita*, determined by densitometric analysis of SDS-R-PAGE patterns.

## References

[1] Kostić M., Smiljković M., Petrović J., Glamočlija J., Barros L., Ferreira I.C.F.R., Ćirić A., Soković M. (2017). Chemical, nutritive composition and a wide range of bioactive properties of honey mushroom: *Armillaria mellea* (Vahl: Fr.) Kummer. Food Funct..

[2] Glamočlija J., Kostić M., Soković M. (2019). Antimicrobial and hepatoprotective activities of edible mushrooms. Biology of Macrofungi.

[3] Kostić M., Ivanov M., Fernandes A., Pinela J., Calhelha R.C., Glamočlija J., Barros L., Ferreira I.C.F.R., Soković M., Ćirić A. (2020). Antioxidant Extracts of Three Russula Genus Species Express Diverse Biological Activity. Molecules.

[4] Yadav D., Negi P.S. (2021). Bioactive components of mushrooms: Processing effects and health benefits. Food Res. Int..

[5] Cateni F., Gargano M.L., Procida G., Venturella G., Cirlincione F., Ferarro V. (2022). Mycochemicals in wild and cultivated mushrooms: Nutrition and health. Phytochem. Rev..

[6] Diyabalanage T., Mulabagal V., Mills G., DeWitt D.L., Nair M.G. (2008). Health-beneficial qualities of the edible mushroom, *Agrocybe aegerita*. Food Chem..

[7] Petrović J., Stojković D., Reis F.S., Barros L., Glamočlija J., Ćirić A., Ferreira C.F.R.I., Soković M. (2014). Study on chemical, bioactive and food preserving properties of *Laetiporus sulphureus* (Bull.: Fr.) Murr. Food Funct..

[8] Petrović J., Glamočlija J., Stojković D., Ćirić A., Barros L., Ferreira C.F.R.I., Soković M. (2015). Nutritional value, chemical composition, antioxidant activity and enrichment of cream cheese with chestnut mushroom *Agrocybe aegerita* (Brig.) Sing. JFST.

[9] Khatua S., Ghosh S., Acharya K. (2017). *Laetiporus sulphureus* (Bull.: Fr.) Murr. as Food as Medicine. Pharmacogn. J..

[10] Cerletti C., Esposito S., Iacoviello L. (2021). Edible Mushrooms and Beta-Glucans: Impact on Human Health. Nutrients.

[11] Roncero-Ramos I., Mendiola-Lanao M., Pérez-Clavijo M., Delgado-Andrade C. (2017). Effect of different cooking methods on nutritional value and antioxidant activity of cultivated mushrooms. Int. J. Food Sci. Nutr..

[12] Sari M., Prange A., Lelley J.I., Hambitzer R. (2017). Screening of beta-glucan contents in commercially cultivated and wild growing mushrooms. Food Chem..

[13] Glamočlija J., Stojković D., Nikolić M., Ćirić A., Barros L., Ferreira C.F.R.I., Soković M. (2015). Comparative study on edible *Agaricus* mushrooms as functional foods. Food Funct..

[14] Banik S.P., Pal S., Ghorai S., Chowdhury S., Khowala S. (2009). Interference of sugars in the Coomassie Blue G dye binding assay of proteins. Anal. Biochem..

[15] Masoumi B., Tabibiazar M., Golchinfar Z., Mohammadifar M., Hamishehkar H. (2024). A review of protein-phenolic acid interaction: Reaction mechanisms and applications. Crit. Rev. Food Sci. Nutr..

[16] Alves M.J., Ferreira I.C., Froufe H.J., Abreu R.M., Martins A., Pintado M. (2013). Antimicrobial activity of phenolic compounds identified in wild mushrooms, SAR analysis and docking studies. J. Appl. Microbiol..

[17] Çayan F., Tel-Çayan G., Deveci E., Duru M.E. (2021). A comprehensive study on phenolic compounds and bioactive properties of five mushroom species via chemometric approach. J. Food Process..

[18] Bauer Petrovska B., Panov S., Zafirovska D.R., Kulevanova S. (2004). Electrophoretic study of mushroom proteins. J. Agric. Food Environ..

[19] Prandi B., Cigognini I.M., Faccini A., Zurlini C., Rodríguez Ó., Tedeschi T. (2023). Comparative Study of Different Protein Extraction Technologies Applied on Mushrooms By-products. Food Bioprocess. Technol..

[20] Kurien B.T., Scofield R.H. (2012). Common artifacts and mistakes made in electrophoresis. Methods Mol. Biol..

[21] Tehrani M.H.H., Fakhrehoseini E., Nejad M.K., Mehregan H., Hakemi-Vala M. (2012). Search for Proteins in the Liquid Extract of Edible Mushroom, *Agaricus bisporus*, and Studying their Antibacterial Effects. Iran. J. Pharm. Res..

[22] Valentão P., Lopes G., Valente M., Barbosa P., Andrade P.B., Silva B.M., Baptista P., Seabra R.M. (2005). Quantitation of Nine Organic Acids in Wild Mushrooms. J. Agric. Food Chem..

[23] Valentão P., Andrade P.B., Rangel J., Ribeiro B., Silva B.M., Baptista P., Seabra R.M. (2005). Effect of the Conservation Procedure on the Contents of Phenolic Compounds and Organic Acids in Chanterelle (*Cantharellus cibarius*) Mushroom. J. Agric. Food Chem..

[24] Park Y.J., Jung E.S., Singh D., Lee D.E., Kim S., Lee Y.W., Kim J.-G., Lee C.H. (2017). Spatial (cap & stipe) metabolomic variations affect functional components between brown and white beech mushrooms. Food Res. Int..

[25] Gamboa-Becerra R., Montoya L., Bandala V.M., Monribot-Villanueva J.L., Guerrero-Analco J.A., Ramos A. (2024). Metabolomic profiling, nutritional parameters and potential bioactive metabolites of the edible mushroom *Tricholoma mesoamericanum*. Int. J. Food Sci..

[26] Ye L., Zhang B., Yang X., Li X., Tan W., Zhang X. (2023). Ultra-performance liquid chromatography-tandem mass spectrometry revealed the significantly different metabolic profiles of *Auricularia cornea* growing on weakly acidic and weakly alkaline substrates. Can. J. Microbiol..

[27] Palacios I., Lozano M., Moro C., D’Arrigo M., Rostagno M.A., Martínez J.A., García-Lafuente A., Guillamón E., Villares A. (2011). Antioxidant properties of phenolic compounds occurring in edible mushrooms. Food Chem..

[28] Reis F.S., Martins A., Barros L., Ferreira I.C.F.R. (2012). Antioxidant properties and phenolic profile of the most widely appreciated cultivated mushrooms: A comparative study between in vivo and in vitro samples. Food Chem. Toxicol..

[29] Quintero-Cabello K.P., Palafox-Rivera P., Lugo-Flores M.A., Gaitán-Hernández R., González-Aguilar G.A., Silva-Espinoza B.A., Tortoledo-Ortiz O., Ayala-Zavala J.F., Monribot-Villanueva J.L., Guerrero-Analco J.A. (2021). Contribution of bioactive compounds to the antioxidant capacity of the edible mushroom *Neolentinus lepideus*. Chem. Biodivers..

[30] Kim M.-Y., Seguin P., Ahn J.-K., Kim J.-J., Chun S.-C., Kim E.-H., Seo S.-H., Kang E.-Y., Kim S.-L., Park Y.-J. (2008). Phenolic Compound Concentration and Antioxidant Activities of Edible and Medicinal Mushrooms from Korea. J. Agric. Food Chem..

[31] Nowacka N., Nowak R., Drozd M., Olech M., Los R., Malm A. (2014). Analysis of phenolic constituents, antiradical and antimicrobial activity of edible mushrooms growing wild in Poland. LWT Food Sci. Technol..

[32] Koutrotsios G., Kalogeropoulos N., Kaliora A.C., Zervakis G.I. (2018). Toward an Increased Functionality in Oyster (Pleurotus) Mushrooms Produced on Grape Marc or Olive Mill Wastes Serving as Sources of Bioactive Compounds. J. Agric. Food Chem..

[33] Zhao X., Hengchao E., Dong H., Zhang Y., Qiu J., Qian Y., Zhou C. (2022). Combination of untargeted metabolomics approach and molecular networking analysis to identify unique natural components in wild *Morchella* sp. by UPLC-Q-TOF-MS. Food Chem..

[34] Gonzaga M.L.C., Menezes T.M.F., de Souza J.R.R., Ricardo N.M.P.S., Soares S.d.A. (2013). Structural characterization of β glucans isolated from *Agaricus blazei* Murill using NMR and FTIR spectroscopy. Bioact. Carbohydr. Diet Fibre.

[35] Taofiq O., González-Paramás A.M., Martins A., Barreiro M.F., Ferreira I.C. (2016). Mushrooms extracts and compounds in cosmetics, cosmeceuticals and nutricosmetics—A review. Ind. Crop. Prod..

[36] Majtan J., Jesenak M. (2018). β-Glucans: Multi-Functional Modulator of Wound Healing. Molecules.

[37] Kim J.H., Tam C.C., Chan K.L., Mahoney N., Cheng L.W., Friedman M., Land K.M. (2022). Antimicrobial Efficacy of Edible Mushroom Extracts: Assessment of Fungal Resistance. Appl. Sci..

[38] Moussa A.Y., Fayez S., Xiao H., Xu B. (2022). New insights into antimicrobial and antibiofilm effects of edible mushrooms. Int. Food Res..

[39] Vamanu E., Nita S. (2013). Antioxidant capacity and the correlation with major phenolic compounds, anthocyanin, and tocopherol content in various extracts from the wild edible *Boletus edulis* mushroom. Biomed. Res. Int..

[40] Cheng J.J., Lin C.Y., Lur H.S., Chen H.P., Lu M.K. (2008). Properties and biological functions of polysaccharides and ethanolic extracts isolated from medicinal fungus, *Fomitopsis pinicola*. Process Biochem..

[41] Bradford M.M. (1976). A rapid and sensitive method for the quantitation of microgram quantities of protein utilizing the principle of protein-dye binding. Anal. Biochem..

[42] Milinčić D.D., Kostić A.Ž., Gašić U.M., Lević S., Stanojević S.P., Barać M.B., Tešić Ž.L., Nedović V., Pešić M.B. (2021). Skimmed Goat’s Milk Powder Enriched with Grape Pomace Seed Extract: Phenolics and Protein Characterization and Antioxidant Properties. Biomolecules.

[43] Milinčić D.D., Stanisavljević N.S., Kostić A.Ž., Soković Bajić S., Kojić M.O., Gašić U.M., Barać M.B., Stanojević S.P., Tešić Ž., Pešić M.B. (2021). Phenolic compounds and biopotential of grape pomace extracts from Prokupac red grape variety. LWT Food Sci. Technol..

[44] Pesic M.B., Barac M.B., Stanojevic S.P., Ristic N.M., Macej O.D., Vrvic M.M. (2012). Heat induced casein-whey protein interactions at natural pH of milk: A comparison between caprine and bovine milk. Small Ruminant Res..

[45] Milinčić D.D., Vidović B.B., Gašić U.M., Milenković M., Kostić A.Ž., Stanojević S.P., Ilić T., Pešić M.B. (2024). A systematic UHPLC Q-ToF MS approach for the characterization of bioactive compounds from freeze-dried red goji berries (*L. barbarum* L.) grown in Serbia: Phenolic compounds and phenylamides. Food Chem..

[46] Pešić M.B., Milinčić D.D., Kostić A.Ž., Stanisavljević N.S., Vukotić G.N., Kojić M.O., Gašić U.M., Barać M.B., Stanojević S.P., Popović D.A. (2019). In vitro digestion of meat- and cereal-based food matrix enriched with grape extracts: How are polyphenol composition, bioaccessibility and antioxidant activity affected?. Food Chem..

[47] Stojković D., Dias M.I., Drakulić D., Barros L., Stevanović M., Ferreira I.C.F.R., Soković M. (2020). Methanolic Extract of the Herb *Ononis spinosa* L. Is an Antifungal Agent with no Cytotoxicity to Primary Human Cells. Pharmaceuticals.

[48] Stojković D., Kovačević-Grujičić N., Reis F., Davidović S., Barros L., Popović J., Petrović I., Pavić A., Glamočlija J., Ćirić A. (2017). Chemical composition of the mushroom *Meripilus giganteus* Karst. and bioactive properties of its methanolic extract. LWT Food Sci. Technol..

[49] CLSI (2009). Clinical and Laboratory Standards Institute Methods for Dilution Antimicrobial Susceptibility Tests for Bacteria that Grow Aerobically. Approved Standard.

[50] Kostić M., Ivanov M., Babić S.S., Petrović J., Soković M., Ćirić A. (2020). An up-to-date review on bio-resource therapeutics effective against bacterial species frequently associated with chronic sinusitis and tonsillitis. Curr. Med. Chem..

