# Supplementary material for: Comparative Chemical Analysis and Bioactive Properties of Aqueous and Glucan-Rich Extracts of Three Widely Appreciated Mushrooms: Agaricus bisporus (J.E.Lange) Imbach, Laetiporus sulphureus (Bull.) Murill and Agrocybe aegerita (V. Brig.) Vizzini
Source: Pharmaceuticals (Basel). 2024 Aug 31;17(9):1153. doi: 10.3390/ph17091153 (PMC11434720; doi:10.3390/ph17091153)
Supplement: Supplementary file 1 [file pharmaceuticals-17-01153-s001.zip › pharmaceuticals-3151662-supplementary.pdf]

Supplementary Materials:

Table S1. Polypeptide composition (%) of mushroom aqueous extract of *A. bisporus* and *A. aegerita*, determined by densitometric analysis of SDS-R-PAGE patterns.

	A.bisporus			*A.aegerita*		
MW ranges	No. band	MW (kDa)	%	No. band	MW (kDa)	%
				1	177.1	2.07±0.13
	1	174.8	3.29±0.16			
				2	132.3	1.00±0.09
>66.2	2	94.8	0.72±0.00	3	113.5	1.74±0.00
				4	86.1	1.60±0.28
	3	82.5	0.85±0.07			
	4	74.3	1.84±0.11			
				5	73.8	2.32±0.04
total			6.70			8.73
	5	60.3	1.89±0.19			
				6	58.4	1.82±0.19
	6	56.3	1.87±0.11			
	7	53.6	1.10±0.21			
				7	46.6	1.82±0.04
35-66.2	8	46.3	2.21±0.02			
	9	41.8	1.83±0.06			
				8	39.5	2.39±0.27
				9	38.5	1.75±0.05
	10	37.0	1.96±0.02			
total			10.86			7.78
	11	34.5	2.22±0.01			
				10	29.4	1.36±0.27
	12	28.5	3.39±0.15			
	13	25.7	2.53±0.14			
	14	24.2	4.40±0.17			
				11	22.7	6.64±0.38
14.4-35	15	19.9	5.29±0.09			
				12	19.7	22.88±0.80
				13	18.9	9.61±0.39
	16	18.5	5.08±0.11			
				14	17.9	10.89±0.49
	17	17.6	7.49±0.19			
	18	17.2	13.82±1.34		/	/
				15	16.9	7.10±0.00
total			44.22			58.48
<14.4	19	<14.4	38.21±0.83	16	<14.4	18.37±0.10
				17	<14.4	6.64±0.71
total			38.21			25.01
